# Subcutaneous foslevodopa in akinetic crisis. A case report from the neurological intensive care unit

**DOI:** 10.3389/fmed.2024.1446345

**Published:** 2024-08-26

**Authors:** Moritz A. Loeffler, Annerose Mengel, Constanze Single, Daniel Weiss, Katharina Feil

**Affiliations:** ^1^Centre for Neurology, Department for Neurodegenerative Diseases, University of Tuebingen, Tuebingen, Germany; ^2^Hertie-Institute for Clinical Brain Research, University of Tuebingen, Tuebingen, Germany; ^3^Centre for Neurology, Department for Vascular Neurology, University of Tuebingen, Tuebingen, Germany

**Keywords:** akinetic crisis, foslevodopa, hydrocephalus, Parkinson, case report

## Abstract

**Introduction:**

Akinetic crisis is a severe deterioration of motor performance occurring in syndromes with pre- or postsynaptic dopaminergic deficit, necessitating effective dopamine replacement therapy. The subcutaneously applicable levodopa derivative foslevodopa represents a new therapeutic option for patients with advanced Parkinson’s disease as a continuous therapy. However, its potential role as a parenteral treatment option for akinetic crisis has not been investigated, yet.

**The case:**

A 78 year-old patient who had developed akinetic-rigid symptomatology in the context of normal pressure hydrocephalus was admitted to our intensive care unit after experiencing an acute exacerbation of akinesia in the context of pulmonary infection. Off-label administration of subcutaneous foslevodopa was initiated after repeated failures to insert a gastric tube for enteral application of levodopa and contraindications against amantadine and apomorphine.

**Results:**

Following the administration of a subcutaneous test dose, continuous application of foslevodopa via a B. Braun syringe pump was gradually increased to 0.3 mL/h during the daytime and 0.15 mL/h at night, corresponding to a levodopa equivalent dosage of 1,020 mg/d. This was accompanied by an improvement of the MDS-UPDRS III score from 85 points to 59 points after 72 h.

**Discussion:**

Treatment of an akinetic crisis with subcutaneous foslevodopa in an intensive care unit setting has proven to be safe and effective in a patient with acute akinesia associated with dopamine-sensitive hydrocephalus. Due to the pathophysiological distinction from Parkinson’s disease, there may be differences in therapeutic response and side effects. Nevertheless, the method used here can serve as a protocol basis for the treatment of akinetic crises with foslevodopa in general and as a starting point for further research.

## Introduction

Akinetic crises, characterised by a severe and acute exacerbation of motor performance, occur in clinical syndromes associated with pre- or postsynaptic dopaminergic deficit. Akinetic crisis is a medical potentially life-threating emergency that often requires intensive care unit (ICU) admission due to the high risk of complications such as respiratory failure, dysphagia, aspiration and severe autonomic dysfunction—especially if not promptly diagnosed and treated. Akinetic crises may be triggered by infections, surgery, trauma, or the withdrawal of dopaminergic treatment. Distinct from typical dopaminergic motor fluctuations, akinesia may persist despite the administration of dopaminergic medication for more than 48 h (1). The management of these patients is particularly challenging due to the need for rapid stabilisation and the management of concurrent medical issues. ICU treatment protocols typically focus on supportive care, optimisation of dopaminergic therapy, and addressing the underlying triggers of the crisis. Traditional treatments for akinetic crisis include several approaches, each with its own limitations: (i) enteral levodopa administration via gastric tube reliant on enteral absorption, which can be compromised in critically ill patients or those with gastrointestinal hypomotility, (ii) intravenous amantadine administration, limited by anticholinergic neuropsychiatric side effects such as confusion and hallucinations and QTc interval prolongation, which can be particularly problematic in patients with preexisting cardiovascular issues, and (iii) continuous subcutaneous apomorphine, hindered by similar neuropsychiatric and vegetative side effects, such as severe nausea and hypotension, that can delay rapid dose titration to overcome the akinetic state (2).

Foslevodopa is a recently approved hydrophilic levodopa derivate, for continuous dopamine replacement therapy in patients with advanced Parkinson’s Disease (PD) and motor fluctuations. Foslevodopa’s hydrophilic nature facilitates its continuous infusion, providing more stable plasma levels of levodopa and thereby improving motor control without the peaks and troughs associated with oral levodopa administration (3, 4). The subcutaneous administration could be of particular advantage in the ICU setting, where enteral routes may be compromised and intravenous routes may not apply for long-term dopaminergic therapy. Yet, its efficacy in the intensive care unit (ICU) management of akinetic crisis remains unexplored. This case report aims to address this gap by presenting the outcomes of foslevodopa administration in a patient with akinetic crisis associated with normal pressure hydrocephalus. The case underscores the challenges of managing akinetic crises and explores the potential role of foslevodopa as a therapeutic option, highlighting the need for further research in this area.

### Case description

Here, we report the case of a 78 year-old male patient admitted to our neurological ICU with diminished consciousness and acutely exacerbated parkinsonism. The clinical presentation was marked by generalised severe cogwheel rigidity, akinesia, rest tremor, anarthria, and dysphagia. His pronounced neck rigidity was initially interpreted as meningism. Accompanying symptoms included fever up to 38.6°C and severe hypotension (mean arterial blood pressure of 50–60 mmHg). The patient had a history a normal pressure hydrocephalus diagnosed at the age of 55 years, and characterised by the typical symptomatology of urinary urge incontinence, mnestic deficits, and gait disorder. There was no imaging evidence of aqueductal stenosis. At the age of 65, he underwent ventriculoperitoneal shunting, which led to symptom alleviation. Noteworthy, his clinical condition had deteriorated over the last months before acute admission manifesting as progressively worsening generalised parkinsonism of the lower but also upper body leading to progressive immobility and an impairment of fine motor skills relevant to everyday life necessitating relocation to a nursing home. In the 2 weeks before admission, the patient was not mobile at all leading to deep vein thrombosis. Dopaminergic medication had not been administered during this period.

**Figure 1:**
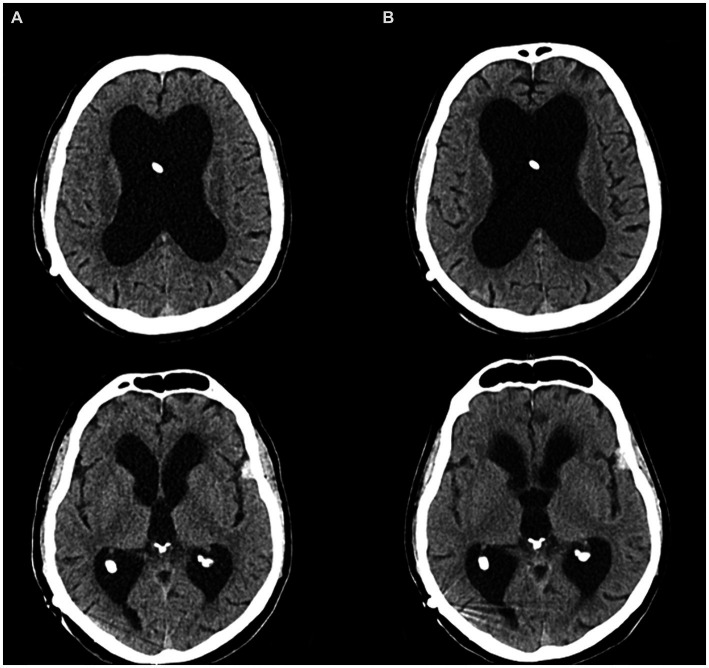
CT imaging of the vetricular system. **(A)** Routine CT scan 8 months prior to the akinetic crisis. **(B)** CT scan directly before admission to the intensive care unit.

Upon admission, given the patient’s history of a shunted hydrocephalus, we initially suspected either a shunt dysfunction leading to increased intracranial pressure or a shunt infection causing ventriculitis and potentially meningoencephalitis. However, cranial computer tomography (CT) revealed no evidence of shunt dysfunction, as ventricular morphology remained consistent with the previous CT scan from 7 months ago (Figure 1). Furthermore, a lumbar puncture showed a normal cerebrospinal fluid (CSF) cell count and protein level, effectively excluding a shunt infection. Laboratory diagnostics indicated a pronounced inflammatory response with leukocytosis and elevated levels of c-reactive protein (CRP) and procalcitonin (PCT). Consequently, empirical antibiotic therapy with piperacillin/tazobactam was initiated. To identify the source of infection, whole-body CT imaging was conducted, which ultimately identified pneumonia as the underlying infectious condition.

### Diagnostic assessment and therapeutic intervention of akinesia

**Figure 2:**
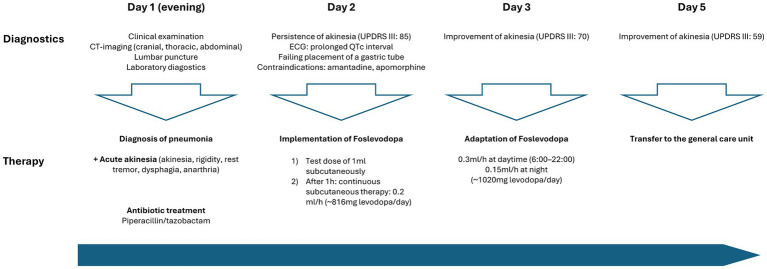
Timeline of patient care and implementation dopaminergic treatment at the intensive care unit.

Against the backdrop of the infection, the patient experienced an acute and severe neurological deterioration of motor performance, manifesting as generalised parkinsonism. This presentation was characterised by typical features such as cogwheel rigidity, rest tremor, bradykinesia, hypokinesia and dysphagia quantified by an *Unified Parkinson’s Disease Rating scale* (MDS-UPDRS III) of 85 points. Additionally, serum creatine kinase levels were elevated at 1070 U/L. Consequently, we evaluated this condition as an akinetic crisis triggered by the infection, necessitating the immediate implementation of dopaminergic treatment (Figure 2).

The initial approach of enteral administration of levodopa was unsuccessful due to severe dysphagia and repeated failures to insert a gastric tube. Amantadine was not considered due to a prolonged QTc interval (488 ms), which posed a significant risk of cardiac arrhythmias. Furthermore, subcutaneous apomorphine was not considered due to the patient’s prolonged hypotension during the infection, requiring for the administration of catecholamines.

Due to the above-mentioned contraindications against the standard therapeutic agents in akinetic crisis, we explored alternative treatment options. Based on our prior experience in using foslevodopa in patients with advanced PD, we decided to initiate off-label use of subcutaneous foslevodopa in our ICU setting. After administering a test dose of 1 mL foslevodopa subcutaneously (equivalent to 170 mg levodopa) without immediate side effects or a clear clinical improvement of motor performance within 1, continuous treatment was commenced using a B. Braun syringe pump, the same system as used in the phase 1 trial (3).

**Figure 3:**
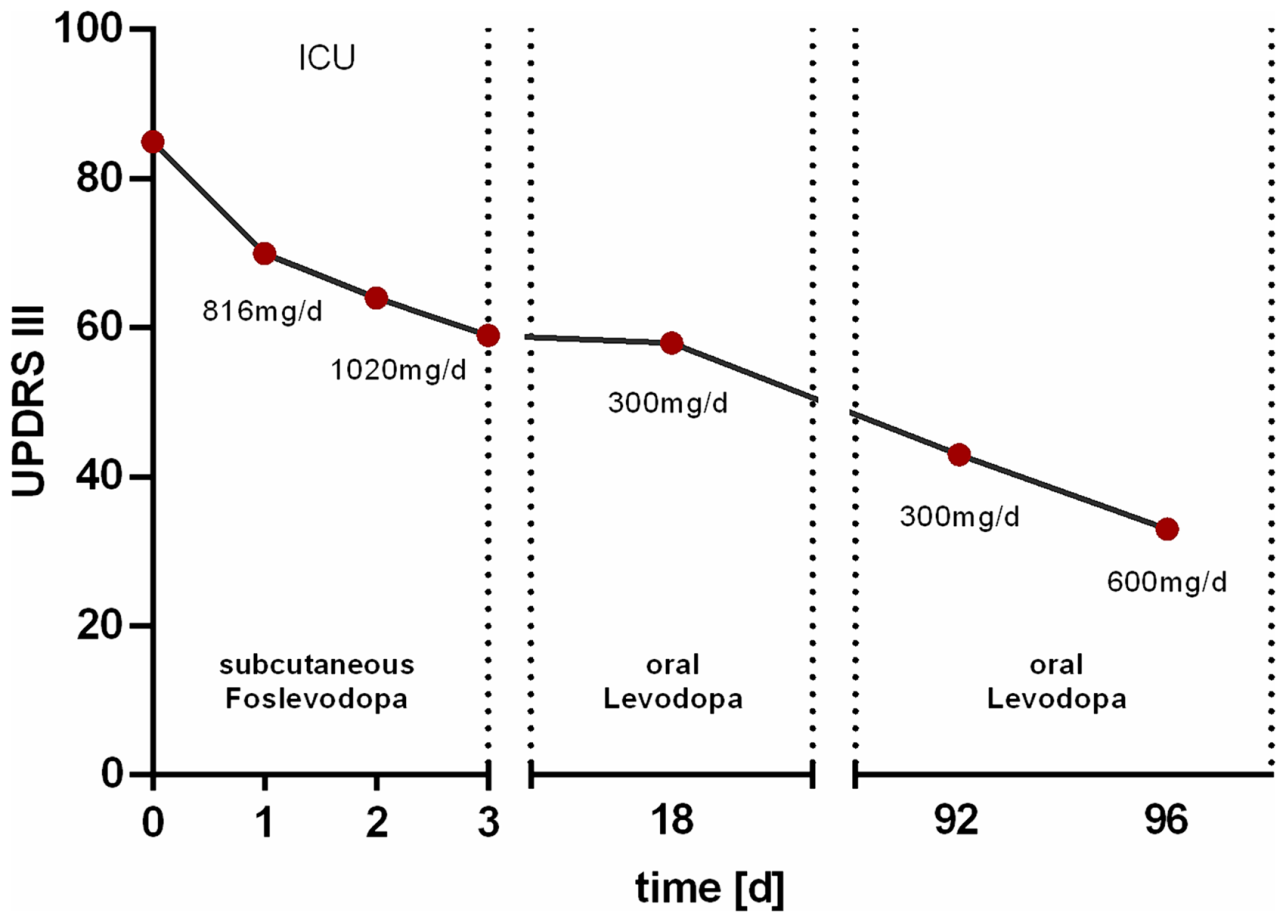
Change in motor performance assessed by the MDS-UPDRS III score. The Unified Parkinson’s Disease Rating Scale (MDS-UPDRS III) score was initially 85 points prior to the subcutaneous administration of 816 mg/day foslevodopa in the intensive care unit (ICU). After 1 day of treatment, the score decreased to 70 points. Upon increasing the dose to 1,020 mg/day, the score further decreased to 64 points after 2 days and 59 points after 3 days. At discharge, 18 days after the initiation of therapy, the score was 58 points under oral medication with 300 mg/day levodopa. At a follow-up 92 days after therapy initiation, the MDS-UPDRS III score was 43 points, which further decreased to 33 points following an increase in the levodopa dose to 600 mg/day.

**Figure 4:**
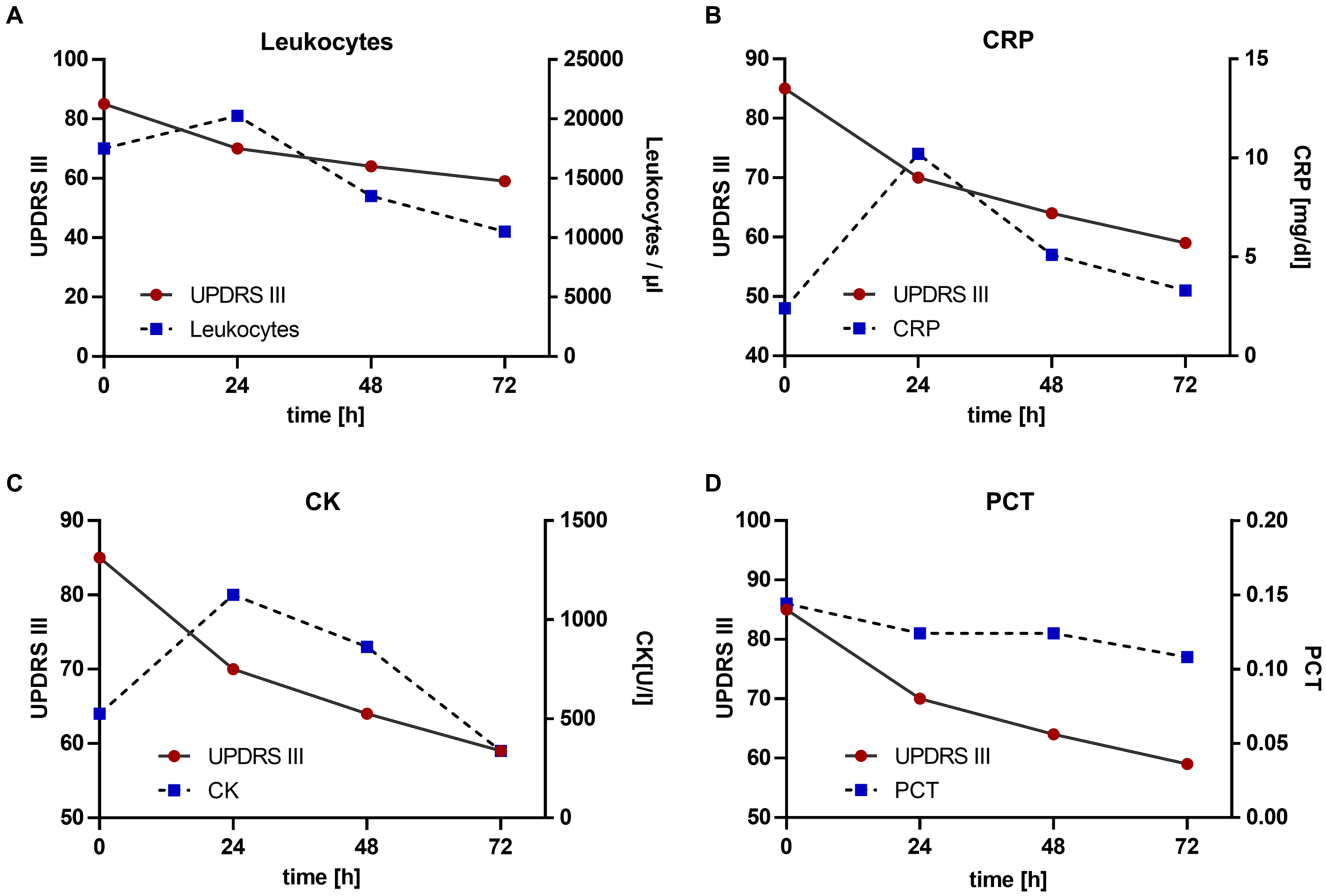
Change in motor performance with reference to laboratory inflammation parameters. Antibiotic treatment was started 15 h prior to the initiation of foslevodopa. The values are given since the initiation of foslevodopa. **(A)** Leukocytes/μL (0 h: 19.330/μL, 24 h: 22.480/μL, 48 h: 14.960/μL, 72 h: 11640/μL), standard value <10.300/μL. **(B)** C-reactive protein (CRP) (0 h: 8.04 mg/dL, 24 h: 12.37 mg/dL, 48 h: 9.45 mg/dL, 72 h: 8.54 mg/dL), standard value <0.50 mg/dL. **(C)** Creatine kinase (CK) (0 h: 1070 U/L, 24 h: 1332 U/L, 48 h: 1220 U/L, 72 h: 990/μL), standard value <190 U/L. **(D)** Procalcitonin (PCT) (0 h: 0.19, 24 h: 0.18, 48 h: 0.18, 72 h: 0.17), standard value <0.1.

The continuous subcutaneous infusion started at 0.2 mL/h, providing a dosage of 816 mg levodopa/day. After 24 h, the dose was adjusted to 0.3 mL/h at daytime (6:00–22:00) and tapered to 0.15 mL/h at night, corresponding to a levodopa equivalent daily dose (LEDD) of 1,020 mg. Over the next 72 h, we observed a significant improvement of the patient’s akinetic-rigid symptoms. The resting tremor ceased completely. This clinical improvement was reflexed by a reduction of the MDS-UPDRS III score from 85 to 59 points 72 h after foslevodopa initiation (Figure 3). The patient’s swallowing and speaking abilities were also restored. On the second day after initiation of dopaminergic treatment, a decline in laboratory inflammation parameters was observed confirming the effectiveness of the antibiotic treatment which was started 15 h before foslevodopa (Figure 4). After 3 days of subcutaneous foslevodopa, transition to oral dopaminergic medication was possible and the patient was transferred to the general care unit. As the infection subsided under antibiotic treatment during the following days, the dopaminergic medication was gradually tapered. To further evaluate the underlying cause of the akinetic crisis in our patient, whether related to the pre-existing normal pressure hydrocephalus or an independent neurodegenerative condition like PD, explaining the slowly progressive onset of akinesia in the year prior to the manifestation of the akinetic crisis, we conducted an ioflupane-PET (DaTSCAN) revealing no evidence of deficiency of presynaptic dopamine transporters.

The patient was discharged 18 days after the initiation of therapy with a LEDD of 300 mg/d showing sustainingly improved motor performance. Upon discharge, the patient’s motor performance, −particularly in the upper extremities, had improved significantly enabling him to eat and dress independently which had not been possible in the pre-hospitalisation state without dopaminergic treatment. During a 3 month follow-up period, motor performance, particularly with regard to leg mobility, improved further which was reflected in an MDS-UPDRS III score of 43 points. Following an increase in LEDD to 600 mg/d during this follow-up visit, further improvements in limb mobility and almost complete resolution of rigidity were observed, reflected in an MDS-UPDRS III score of 33 points (Figure 3).

## Discussion

This case demonstrated that subcutaneous foslevodopa is manageable in an ICU setting for the treatment of akinetic crisis. Alongside the anti-infective treatment, subcutaneous foslevodopa contributed in the restitution of our patient’s motor performance without causing neuropsychiatric side effects often observed with amantadine or apomorphine, even though dopaminergic stimulation was rapidly increased from no medication to a LEDD of around 1,000 mg/day.

The fact that akinetic crises generally occur in patients with PD characterised by presynaptic dopaminergic deficit which was ruled out by DaTSCAN is a limitation concerning the generalisation of this case. However, the negative DaTSCAN result does not negotiate a contribution of dopaminergic transmission to the observed improvements in our patient. Prospective analyses of adult hydrocephalus patients demonstrated that 88 of 118 patients showed additional akinetic or tremulous movement disorders primarily but not exclusively attributed to other co-manifesting neurodegenerative causes (5). Further, in hydrocephalus patients with a regular presynaptic dopaminergic system, a postsynaptic D2 receptor deficit was observed (6), which can be temporally and conditionally variable within a patient (7) and may explain the acute hypodopaminergic state that was triggered by the infection in our case. Further, dopamine replacement therapy was described to be effective in some patients with idiopathic hydrocephalus (8). In the context of severe infection and inflammation, cytokines like interferon alpha can decrease dopamine release and D2 receptor binding at the nigrostriatal synapse (9). In addition, electrolyte shifts and cytokines reduce dopamine uptake through the blood–brain barrier (10). These mechanisms may lead to striatal dopamine deficiency triggering akinetic crisis (11) as observed in our case.

The rapid clinical improvement in motor function following the administration of subcutaneous foslevodopa and preceding the decline in laboratory inflammation parameters supports the effect of dopamine replacement therapy alongside anti-infective and supportive care contributions. However, based on these clinical observations we cannot ultimately ascertain to which extent the dopaminergic therapy was responsible for overcoming the akinetic crisis apart from the anti-infective therapy.

The observation, that at three months follow-up, a further dose increase of levodopa led to a further improvement of the MDS-UPDRS III score underlines dopamine responsiveness of his Parkinsonism. Dopaminergic treatment enabled the patient to dress and eat independently and to exercise walking due to improved leg mobility. None of this has been possible prior to the infection without dopaminergic treatment.

Furthermore, relevant differences to the treatment of an akinetic crisis in PD should be considered: In Parkinson’s disease, which is characterised by a presynaptic dopaminergic deficit, a better dopaminergic responsiveness of motor performance to foslevodopa is conceivable as foslevodopa is metabolised to levodopa by the alkaline phosphatase (3), enabling stimulation of regularly expressed postsynaptic D1 and D2 receptors (12, 13). As PD patients are prone to develop neuropsychiatric side effects as part of their non-motor symptomatology trigged by dopaminergic stimulation (14) they may also be more likely to experience hallucinations under foslevodopa treatment.

Moreover, our patient had not taken any dopaminergic medication before the onset of the akinetic crisis necessitating an estimated LEDD based on experiences with PD patients and adjustments according to the clinical response. In PD patients, the starting dose of continuous foslevodopa could be calculated based on the LEDD of the last oral dopaminergic medication taken before the crisis.

In summary, this case highlights the importance of prompt recognition and innovative management of akinetic crisis in the setting of an ICU to prevent further complications and critical outcomes.

Management of akinetic crises in the ICU involves a multidisciplinary approach including neurologists, intensivists and nursing staff to monitor and adjust treatment promptly. Subcutaneous foslevodopa may be considered as an option for parenteral dopamine replacement therapy with the advantage of improved bioavailability and a potentially favourable side effect profile. However, the acute modification or introduction of dopaminergic treatment in severely affected and vulnerable patients at the intensive care unit always requires for extensive assessment and consideration.

In this context, the novel therapeutic possibility using subcutaneous foslevodopa in an ICU setting warrants further investigation through prospective observational studies to enhance care for severe neurocritical conditions like akinetic crisis.

## Data Availability

The raw data supporting the conclusions of this article will be made available by the authors, without undue reservation.
